# Trends of Sepsis Hospitalizations Among Female Active Component U.S. Service Members, 2011–2022

**Published:** 2025-05-01

**Authors:** Alexis A. McQuistan, Michael T. Fan, Sithembile L. Mabila

**Affiliations:** Armed Forces Health Surveillance Division, Public Health Directorate, Defense Health Agency, Silver Spring, MD

## Abstract

Studies of sepsis within the U.S. military population have consistently shown that rates of sepsis have increased over time. The observed higher incidence of sepsis in studies among women compared to men of the active component U.S. military population is of concern and warrants further evaluation, as it diverges from incidence typically observed in the U.S. general population. The objectives of this study were to examine cases of sepsis among active component U.S. service women between January 1, 2011 and December 31, 2022, compare them to active component men in the U.S. military, and identify factors associated with sepsis among female active component service members. In this study, female active component service members evinced higher rates (66.5 per 100,000 person-years) compared to males (36.7 per 100,000 person-years), with a rate of sepsis 1.9 times higher after adjusting for demographic and military-related factors. Rates of sepsis were higher among women with a history of co-morbidities.

Each year, approximately 1.7 million adults in the U.S. develops sepsis, and at least 250,000 adults who develop sepsis die. ^1^ It is estimated that 30-50% of hospitalization deaths are attributable to sepsis, ^1 , 2^ and this estimate increases with increased disease severity. ^3^ In a study of 750 million U.S. hospitalizations over a 22-year period, the rate of sepsis increased from 82.7 to 240.4 cases per 100,000 population from 1979 to 2000. ^4^ From 2016 to 2019 the number of inpatient stays in the U.S. increased by 20.1% to 2.1 million, and with the emergence of COVID-19, the number increased again to 2.5 million. ^5^ Sepsis imposes a great burden on the U.S. health care system, costing an estimated $41.5 billion per year. ^6^ Implementing and maintaining hospital sepsis prevention programs associated with reductions in mortality, lengths of hospital stay, and health care costs is imperative. ^1 , 7^

Studies of sepsis among the U.S. military population have consistently shown increasing rates of sepsis over time. A 2013 study of sepsis in the active component U.S. military population from 2000 to 2012 found an overall incidence of 13.2 cases per 100,000 person-years (p-yrs) and a 570% increase between 2004 and 2012. Definitions and coding practices for sepsis changed during that period, which may have driven the large increase. ^8^ A follow-up study in 2021 found that overall incidence among the active component was 39.8 per 100,000 p-yrs, and annual incidence of sepsis hospitalization increased by 64% from 2011 to 2019. ^9^

Both the 2013 and 2021 studies presented evidence to suggest that sepsis rates were higher among women compared to men in the U.S. military, which is the inverse of trends seen in the general U.S. population. ^1 , 4^ Neither study, however, was able to explain the reason for this difference. The 2021 study noted that, although infections specific to female service members were seen, the numbers were not large enough to account for the differences. ^9^ It also suggested that clinical variability in coding practices could be a factor if, for example, obstetricians were more likely than internal medicine physicians to diagnose sepsis among patients under their care.

**What are the new findings?**

Rates of sepsis hospitalizations among female active component service members have consistently been higher compared to male active component members. Female active component members had 1.9 times higher rates for hospitalization for sepsis compared to active component service men after adjusting for demographic and military-related factors.

**What is the impact on readiness and force health protection?**

Sepsis is a life-threatening condition that is costly to treat. Patients who survive sepsis may experience lasting impacts, such as long-term disability. Recovery from sepsis could lead to lost duty time, disability, or attrition among service members if the infection is severe. Identifying risk factors and prevention measures to reduce sepsis incidence and severity would improve force health protection.

The observed higher incidence of sepsis in previous studies among women of the active component U.S military population is a concern that needs to be further evaluated. The previous studies raised questions about a possible growing threat to women's health in the military due to sepsis. The objectives of this study were to examine cases of sepsis between 2011 and 2022 among active component U.S. service women, compare them to active component men in the U.S. military population, and identify factors associated with sepsis among female active component service members.

## Methods

This retrospective analysis included active component service members (ACSMs) from the Army, Air Force, Navy, and Marine Corps of the U.S. Armed Forces from January 1, 2011 through December 31, 2022. Data to evaluate sepsis were obtained from the Defense Medical Surveillance System (DMSS), the central repository of medical data for service members. DMSS collects inpatient administrative health records for care received at military hospitals and clinics as well as private sector care purchased through TRICARE. Demographic data obtained from DMSS included sex, age category, race and ethnicity, service branch, occupation, geographic region, deployed status, and military rank; race and ethnicity were self-reported. Deployment status was determined by service member deployment at time of sepsis diagnosis or deployment within the prior 30 days. Deployments to unknown locations or bodies of water were not included.

International Classification of Diseases, 9th Revision (ICD-9) and 10th Revision (ICD-10) diagnostic codes were used to define incident cases of sepsis **(Table 1)** . Septicemia was no longer listed in ICD coding following the October 2015 change to ICD-10 codes, with specific codes including causative organisms provided instead. A case was defined as any hospitalization record with a sepsis diagnostic code in any diagnostic position. A 14-day gap in care incidence rule was applied; an individual could be included as a case more than once if more than 14 days elapsed between dates of consecutive incident case-defining encounters. Co-occurring conditions at time of sepsis hospitalization were identified by ICD-9 and ICD-10 codes from the incident sepsis encounter.

**TABLE 1.** Frequency of Case-Defining ICD Codes for Sepsis by Sex, Active Component U.S. Service Members, 2011–2022

ICD Version	ICD Code	Description	Females		Males	
			No.	%	No.	%
ICD-9-coded encounters (2010–September 2015)	003.1	Salmonella septicemia	1	0.1	5	0.1
	022.3	Anthrax septicemia	0	0.0	0	0.0
	038 *	Septicemia	447	46.6	1,767	49.0
	995.91	Sepsis	340	35.4	1,199	33.3
	995.92	Severe sepsis	80	8.3	392	10.9
	785.52	Septic shock	45	4.7	220	6.1
	998.02	Post-operative septic shock	0	0.0	6	0.2
	112.5	*Candida* sepsis	2	0.2	15	0.4
	Female-specific					
	670.2 *	Puerperal sepsis	45	4.7	0	0.0
ICD-10-coded encounters (October 2015–2022)	A02.1	Salmonella sepsis	1	0.1	1	0.0
	A22.7	Anthrax sepsis	0	0.0	0	0.0
	A26.7	Erysipelothrix sepsis	0	0.0	0	0.0
	A32.7	*Listeria* sepsis	0	0.0	1	0.0
	A40 *	Streptococcal sepsis	32	2.2	140	3.8
	A41 *	Other sepsis	1,003	70.2	2,889	77.4
	A42.7	Actinomycotic sepsis	0	0.0	0	0.0
	A54.86	Gonococcal sepsis	4	0.3	3	0.1
	B37.7	*Candida* sepsis	2	0.1	12	0.3
	R65.2 *	Severe sepsis	202	14.1	675	18.1
	T81.12	Post-procedural septic shock	1	0.1	8	0.2
	Female-specific					
	O03 *	Sepsis following spontaneous abortion	52	3.6	0	0.0
	O04.87	Sepsis following induced termination of pregnancy	5	0.4	0	0.0
	O07.37	Sepsis following failed attempted termination of pregnancy	6	0.4	0	0.0
	O08.82	Sepsis following ectopic and molar pregnancy	6	0.4	0	0.0
	O85	Puerperal sepsis	97	6.8	4	0.1
	O86.04	Sepsis following obstetric procedure	18	1.3	0	0.0

* Asterisk indicates that any subsequent digit or character is included.

Abbreviations: ICD, International Classification of Diseases; No., number.

Additional encounter data for pregnancies and co-morbidities prior to sepsis diagnosis were also included **(Table 2)** . Frequencies of sepsis cases among women who were pregnant within 280 days preceding their incident sepsis encounter with a history of a listed comorbidity are described. Pregnancy-related encounters were defined using ICD-9/ICD-10 diagnosis codes **(Table 2)** in any diagnostic position during an inpatient, outpatient, or in-theater medical encounter or with a positive laboratory test result for human chorionic gonadotropin. Co-morbidities were identified based on prior associations with risk of sepsis and mortality risk from sepsis. ^1 , 10 - 12^ Co-morbidity encounters were defined using ICD-9/ICD-10 diagnosis codes in any diagnostic position from an inpatient, outpatient, or in-theater medical encounters. Co-morbidities were assessed for a period of 365 days preceding the incident sepsis date for sepsis cases and prior history of co-morbidity among all female ACSMs. Each co-morbidity was evaluated separately.

**TABLE 2.** Pregnancy-related and Co-Morbidity Diagnosis Codes

Diagnosis	ICD-9 Codes	ICD-10 Codes
Pregnancy and childbirth	V22.0–V22.2,V23. * ,V27. * , V72.42, 630 * –679 *	Z33 * , Z34 * ,Z37 * , Z32.01, O * ,
Immune-compromising conditions	042, 279 * , 280 * -289 * , V42 * , V58.65, 696 * , 277.3 * , 714.0 * -714.3 * , 555 * , 556 * , 558 *	B20, D55 * -D77 * , D80 * -D89 * , Z94 * , Z795 * , L40 * , M04 * -M08 * , K50 * -K52 *
Chronic kidney disease	583 * , 585 * , 586 * , 590.0 * , 590.1 * , 590.2, 590.8 * , 590.9, 591, 593.3, 593.4, 593.5, 593.7 *	N03 * -N16 * , N18 * -N19 *
Any cardiovascular disease	393 * -457 *	I05 * -I89 * , Z95 *
Hypertension	401 * -405 * , 642 *	I10 * -I16 * , O10 * -O16 *
Neoplasms	140 * -239 *	C00 * -D49 *
Metabolic disease	250 * , 6480 * , V58.67, 240 * -246 * , 264 * -269 * , 277.7	E08 * -E13 * , O24 * , Z794 * , E00 * -E07 * , E50 * -E64 * , E88.81 *
Any lung disease	490 * -519 *	J40 * -J99 *
Chronic lower respiratory disease	490 * -492 *	J40 * -J44 *
Substance use disorders	304 * , 305.1 * -305.9 * , 305.0 * , 303.0 * , 303.9 * , 291.0, 291.81	F10 * -F16 * , F18 * -F19 * , F17 *
Alcohol use disorders	303 *	F10 *
Chronic liver disease	570 * -573 * , 070.22, 070.23, 070.32, 070.33, 070.44, 070.54, 070.59	K70 * -K77 * , B18 *

* Asterisk indicates that any subsequent digit or character is included.

Abbreviation: ICD, International Classification of Diseases.

To further evaluate sepsis incidence among women, body mass index (BMI) data were obtained from the Military Health System Data Repository (MDR) Clinical Data Repository (CDR) Vitals and MDR GENESIS Vitals. BMI was categorized as obese (>=30) and not obese (<30). BMI records within 280 days before or after an ‘O’-coded (encounters related to pregnancy) inpatient or outpatient encounter were excluded. Prior history of co-morbidities related to sepsis was defined as having at least 1 inpatient or outpatient encounter with a co-morbidity diagnosis in any diagnostic position.

Incidence rates were calculated as incident sepsis diagnoses per 100,000 p-yrs. Rates were calculated separately for female and male ACSMs. Rates among female ACSMs with a history of selected co-morbidities were calculated. Person-time contributions for each service member were determined from January 1, 2011 through December 31, 2022. Person-time was censored when a service member left the active component or at the end of the surveillance period. Incidence rate ratios (IRRs) and 95% confidence intervals (CIs) were calculated to compare sepsis rates between female and male ACSMs. An adjusted Poisson regression model (adjusted for race and ethnicity, age, service, grade, occupation) calculated adjusted IRRs comparing rates of sepsis among women and men. All analyses were conducted using SAS-Enterprise Guide (version 8.3).

## Results

From January 1, 2011 through December 31, 2022, 6,588 incident cases of hospitalized sepsis occurred among 573,477 (16.8%) female and 2,841,317 (83.2%) male ACSMs. Female ACSMs accounted for 1,684 (25.6%) incident cases of hospitalized sepsis, while male ACSMs accounted for 4,904 (74.4%) incident cases of hospitalized sepsis between 2011 and 2022.

**Table 1** summarizes the most commonly occurring sepsis diagnoses, stratified by sex. A case could be counted multiple times if the incident encounter featured more than 1 sepsis ICD-9/ICD-10 code. Among ICD-9-coded diagnoses, septicemia and sepsis were the most common diagnoses, accounting for 82% of sepsis-related diagnoses among both female and male sepsis cases. Puerperal sepsis was the only female-specific ICD-9 code and accounted for 4.7% of ICD-9 diagnoses. Among ICD-10-coded encounters, 77.4% of sepsis-related diagnoses among men were coded as “other sepsis” (A41 ^*^ ), which included sepsis due to an unspecified organism (A41.9) or *Escherichia coli* (A41.51), and was the most common diagnosis in this category. The ‘other sepsis’ (A41 ^*^ ) code only accounted for 70.2% of sepsis diagnoses among women. Female-specific sepsis diagnoses accounted for 12.9% of ICD-10 sepsis diagnoses among women. Severe sepsis was diagnosed more frequently among men (18.1%) compared to women (14.1%).

**FIGURE 1.**
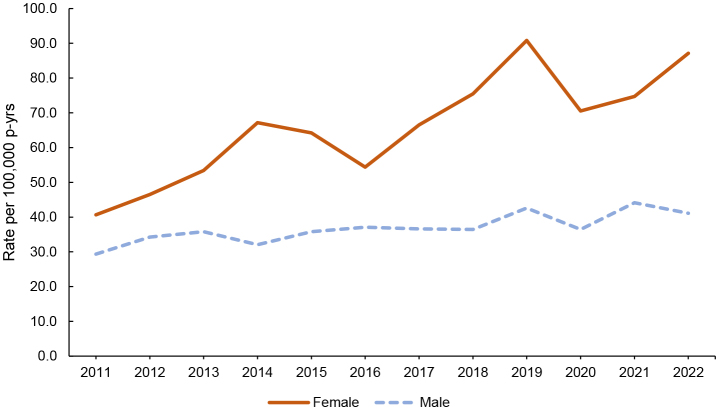
Annual Rates of Sepsis Hospitalizations by Sex, Active Component, U.S. Armed Forces, 2011–2022

**Figure 1** shows the incidence rates of sepsis per 100,000 p-yrs between 2011 and 2022, by sex. Female ACSMs consistently experienced higher rates of sepsis through-out the surveillance period. Among female ACSMs, incidence rates of sepsis peaked in 2019 (90.8 per 100,000 p-yrs) but began to trend upward again after 2020, registering 87.1 per 100,000 p-yrs in 2022. Women in nearly all demographic categories experienced higher rates of sepsis than men **(Table 3)** except in the recruit category, due to the small numbers of women. Crude rate ratios are provided that compare women to men.

**TABLE 3.** Incidence of Sepsis by Sex, Active Component U.S. Service Members, 2011–2022

Demographic Characteristics	Females		Males		Crude Rates		
	No.	Rate ^a^	No.	Rate ^a^	RR	95% LCL	95% UCL
Total	1,684	66.5	4,904	36.7	1.8	1.7	1.9
Age, y							
<20	158	84.6	483	56.1	1.5	1.3	1.8
20–24	630	75.7	1,360	32.3	2.3	2.1	2.6
25–29	349	55.5	932	29.6	1.9	1.7	2.1
30–34	234	58.4	695	32.4	1.8	1.6	2.1
35–39	160	59.9	635	40.2	1.5	1.3	1.8
40–44	86	64.0	427	47.7	1.3	1.1	1.7
45 +	67	81.6	372	72.3	1.1	0.9	1.5
Race and ethnicity							
White, non-Hispanic	712	64.9	3,044	38.0	1.7	1.6	1.9
Black, non-Hispanic	384	60.1	658	34.5	1.7	1.5	2.0
Hispanic	330	74.9	692	35.2	2.1	1.9	2.4
Other	258	72.6	510	34.5	2.1	1.8	2.4
Service branch							
Army	531	62.1	1,824	36.1	1.7	1.6	1.9
Navy	495	67.8	1,051	33.2	2.0	1.8	2.3
Air Force	501	65.4	1,084	35.1	1.9	1.7	2.1
Marine Corps	157	87.2	945	46.1	1.9	1.6	2.2
Rank							
Enlisted	1,479	72.4	4,229	38.3	1.9	1.8	2.0
Officer	205	41.8	675	29.2	1.4	1.2	1.7
Military occupation							
Combat-related	51	83.8	823	37.4	2.2	1.7	3.0
Motor transport	56	72.1	156	40.2	1.8	1.3	2.4
Pilot / air crew	22	58.3	130	23.6	2.5	1.6	3.9
Repair / engineering	346	69.2	1,420	34.0	2.0	1.8	2.3
Communications / intelligence	572	69.1	888	33.9	2.0	1.8	2.3
Health care	299	61.9	366	40.3	1.5	1.3	1.8
Other	338	62.0	1,121	44.5	1.4	1.2	1.6
Recruit status							
Yes	24	43.0	227	82.8	0.5	0.3	0.8
No	1,660	67.0	4,677	35.7	1.9	1.8	2.0
Education level							
High school or less	1,078	74.9	3,331	37.7	2.0	1.9	2.1
Some college	266	71.2	611	39.9	1.8	1.5	2.1
Bachelor's degree or more	318	48.4	880	32.4	1.5	1.3	1.7
Other / unknown	22	34.8	82	28.7	1.2	0.8	1.9
Marital status							
Single	677	59.4	1,943	35.3	1.7	1.5	1.8
Married	812	70.8	2,720	37.0	1.9	1.8	2.1
Other	195	79.4	241	47.8	1.7	1.4	2.0
Deployment status							
Yes	16	24.2	115	21.2	1.1	0.7	1.9
No	1,668	67.6	4,789	37.4	1.8	1.7	1.9

Abbreviations: No., number; RR, rate ratio; LCL, lower confidence limit; UCL, upper confidence limit; y, years.

a Rate per 100,000.

Rates by age group for both sexes displayed a U-shaped pattern in which highest rates were in the under age 20 years (84.6 per 100,000 among females, 56.1 per 100,000 among males) and 45 years and older (81.6 per 100,000 among females; 72.3 per 100,000 among males) age groups in both sexes. The greatest difference between female and male sepsis cases was seen in the 20-24-year age group, in which women were 2.3 times (95% CI, 2.1, 2.6) more likely to have sepsis hospitalizations than men in the same group. Women had higher rates of sepsis compared to men in all racial and ethnic groups.

In every branch of service women also experienced higher rates of sepsis. The highest rates of sepsis for both women and men were in the Marine Corps, with female marines experiencing a rate of 87.2 per 100,000 p-yrs, while males experienced a rate of 46.1 per 100,000 p-yrs. The greatest difference in rates between the sexes was seen in the Navy, with 2.0 times (95% CI, 1.8, 2.3) the rate of sepsis hospitalization among female sailors compared to male sailors.

Among enlisted service members, rates were 1.9 times (95% CI, 1.8, 2.0) higher among women (72.4 per 100,000 p-yrs) than men. Among those in combat-specific occupations, rates were more than twice as high among women then men. Deployed service members had lower rates than those who did not recently deploy, but women had higher rates than men among both groups. Rates of sepsis among female recruits (43.0 per 100,000 p-yrs) were half that of male recruits (82.8 per 100,000 p-yrs). After accounting for the effects of age, race and ethnicity, service, grade, and occupation, the adjusted rate was nearly twice as high among female ACSMs as male ACSMs **(Table 4)** .

**TABLE 4.** Adjusted Incidence Rate Ratios of Sepsis Hospitalizations, Active Component U.S. Service Members, 2011–2022

Demographic Characteristics	Adjusted IRR ^a^	95% CI
Sex		
Female	1.9	1.8–2.0
Male	Reference	
Race and ethnicity		
White, non-Hispanic	Reference	
Black, non-Hispanic	0.9	0.8–0.9
Hispanic	0.9	0.9–1.0
Other	1	0.9–1.0
Age, y		
<20	1.3	1.2–1.5
20–24	0.9	0.9–1.0
25–29	0.9	0.8–1.0
30–34	Reference	
35–39	1.2	1.1–1.4
40–44	1.5	1.4–1.7
45+	2.5	2.2–2.8
Service branch		
Army	Reference	
Navy	1.0	0.9–1.1
Air Force	1.0	0.9–1.1
Marine Corps	1.3	1.2–1.4
Grade		
Enlisted	1.7	1.6–1.9
Officer	Reference	
Military occupation		
Combat-specific	Reference	
Communications / intelligence	0.9	0.8–1.0
Health care	1.1	0.9–1.2
Motor transport	1.0	0.9–1.2
Other	1.1	1.0–1.2
Pilot / air crew	0.8	0.7–1.0
Repair / engineering	0.9	0.8–1.0

Abbreviations: IRR, Incidence rate ratio; CI, confidence interval, y, years.

a Adjusted for sex, age, race and ethnicity, service, grade, occupation

Further descriptions of female sepsis cases are provided in **Tables 5** – **7** . The most frequent co-occurring infections for sepsis cases among female ACSMs are shown in **Table 5** . Among ICD-9-coded encounters, the most common infections were pyelonephritis, pneumonia with unspecified organism, unspecified urinary tract infection (UTI), and post-operative infection. Among ICD-10-coded encounters, the most common infections were acute pyelonephritis, unspecified UTI, COVID-19, unspecified *E. coli* infection, and unspecified pneumonia.

**TABLE 5.** Frequency Distribution of Co-Occurring Infections with Sepsis, Hospitalized Cases, Female Active Component U.S. Service Members, 2011–2022

ICD-9 Code	Description	No.	% of Diagnoses
59080	Pyelonephritis, unspecified	98	2.9
486	Pneumonia, organism unspecified	74	2.2
59010	Acute pyelonephritis without lesion of renal medullary necrosis	56	1.7
5990	UTI, site not specified	51	1.5
99859	Other post-operative infection	32	0.9
4149	Other and unspecified *E. coli*	22	0.6
64783	Other specified infectious and parasitic diseases of mother, antepartum condition or complication	20	0.6
64663	Infections of genitourinary tract In pregnancy, antepartum condition or complication	19	0.6
845	Intestinal infection due to *C. diff* .	11	0.3
340	Streptococcal sore throat	10	0.3

ICD-10 Code	Description	No.	% of Diagnoses
N10	Acute pyelonephritis	182	2.3
N390	Urinary tract infection, site not specified	123	1.6
Z20822	Contact with and (suspected) exposure to COVID-19	103	1.3
B9620	Unspecified *E. coli* as cause of diseases classified elsewhere	98	1.2
J189	Pneumonia, unspecified organism	83	1.1
B9689	Other specified bacterial agents as cause of diseases classified elsewhere	39	0.5
U071	COVID-19	38	0.5
O753	Other infection during labor	27	0.3
O411230	Chorioamnionitis, third trimester, not applicable or unspecified	26	0.3
N739	Female pelvic inflammatory disease, unspecified	25	0.3

Abbreviations: ICD-9, International Classification of Diseases, 9th Revision; No., number; ICD-10, International Classification of Diseases, 10th Revision; UTI, urinary tract infection; *E. coli, Escherichia coli; C. diff., Clostridium difficile* .

**TABLE 6.** Frequency Distribution of Other Co-Occurring Diagnoses with Sepsis of Non-Infectious Conditions, Hospitalized Cases, Female Active Component U.S. Service Members, 2011–2022

ICD-9 Code	Description	No.	% of Diagnoses
2768	Hypopotassemia	63	1.9
5849	Acute kidney failure, unspecified	57	1.7
2859	Anemia, unspecified	45	1.3
2761	Hypo-osmolality and / or hyponatremia	42	1.2
51881	Acute respiratory failure	39	1.2
27651	Dehydration	34	1.0
3051	Tobacco use disorder	34	1.0
5119	Unspecified pleural effusion	31	0.9
591	Hydronephrosis	28	0.8
2762	Acidosis	24	0.7

ICD-10 Code	Description	No.	% of Diagnoses
E876	Hypokalemia	174	2.2
E871	Hypo-osmolality and hyponatremia	130	1.7
N179	Acute kidney failure, unspecified	129	1.6
N12	Tubulo-interstitial nephritis, not specified as acute or chronic	115	1.5
D649	Anemia, unspecified	111	1.4
E872	Acidosis	89	1.1
E860	Dehydration	78	1.0
J9601	Acute respiratory failure with hypoxia	75	1.0
D509	Iron deficiency anemia, unspecified	57	0.7
K5900	Constipation, unspecified	51	0.6

Abbreviations: ICD-9, International Classification of Diseases, 9th Revision; No., number; ICD-10, International Classification of Diseases, 10th Revision.

**TABLE 7.** Numbers and Percentages of Incident Sepsis Cases with Co-Morbidity Encounter, 365 Days Preceding Incident Diagnosis, Female Active Component U.S. Service Members, 2011–2022

Prior Co-Morbidity	No.	%
Pregnancy-related encounter	519	30.8
Immune compromising conditions	517	30.7
Chronic kidney disease	325	19.3
Any cardiovascular disease	315	18.7
Hypertension	200	11.9
Obesity	275	16.3
Neoplasms	271	16.1
Metabolic disease	223	13.2
Any lung disease	223	13.2
Chronic lower respiratory disease	43	2.6
Substance use disorders	200	11.9
Chronic liver disease	85	5.1

Abbreviation: No., number

The most common co-occurring non-infectious diagnoses under ICD-9 coding were hypopotassemia, acute kidney failure, unspecified anemia, hypo-osmolality and / or hyponatremia, and acute respiratory failure **(Table 6)** . Among ICD-10-coded diagnoses, the most common non-infectious diagnoses were hypokalemia, hypo-osmolality and hyponatremia, acute kidney failure, tubule-interstitial nephritis, and unspecified anemia.

The co-occurring diagnoses in **Tables 5** and **6** are limited to those occurring within the incident sepsis encounter. Among female ACSM sepsis cases, 1,187 cases had another health encounter that did not include a sepsis-related diagnosis, within 7 days prior to the incident sepsis hospitalization (data not shown). Seventy-seven cases were hospitalized in the 7 days preceding the incident sepsis encounter, and more than half (n=42, 55%) of those cases had primary diagnoses related to pregnancy, delivery, or postpartum care (data not shown).

Women were also assessed for comorbid conditions in the year preceding sepsis diagnosis, defined as at least 1 diagnosis for a co-morbid condition within 365 days before the incident sepsis diagnosis **(Table 7)** . Just over two-thirds, or 67.2%, of sepsis cases had health care encounters with at least 1 co-morbidity diagnosis (n=1,131), listed in **Table 7** , within 365 days preceding incident their sepsis diagnoses. Immune-compromising conditions were the most common (n=517, 30.7%), followed by chronic kidney disease (n=325, 19.3%), cardiovascular disease (n=315, 18.7%), neoplasms (n=271, 16.1%), and obesity (n=275, 16.3%). Slightly less than one-third, or 30.8%, of women had a pregnancy-related encounter within 280 days preceding an incident sepsis diagnosis.

**FIGURE 2.**
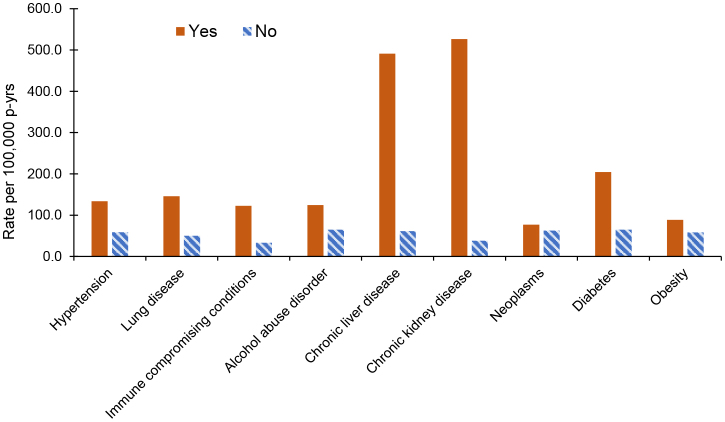
Incidence Rates of Sepsis by Co-Morbidity History, Female Active Component U.S. Service Members, 2011–2022

Rates of sepsis among women with histories of hypertension, lung disease, immune-compromising conditions, alcohol abuse disorders, chronic liver disease, chronic kidney disease, neoplasms, obesity, and diabetes also were evaluated **(Figure 2)** . Rates of sepsis were consistently higher among women with a history of comorbidities, with the highest rates among women with a history of chronic kidney disease (526.3 per 100,000 p-yrs), chronic liver disease (491.2 per 100,000 p-yrs), or diabetes (204.7 per 100,000 p-yrs).

## Discussion

Between January 1, 2011 and December 31, 2022, female ACSMs experienced higher rates of sepsis compared to male ACSMs, consistent with previous studies of the U.S. military population. ^8 , 9^ This difference in rates was true for all demographic groups analyzed except the recruit population. After adjusting for other demographic and military-related factors, female ACSMs still demonstrated higher rates than male ACSMs. While the difference in rates between sexes persists after adjustment, it cannot be ruled out that changes in 2016 in the clinical definitions of sepsis under Sepsis-3 may have had a greater impact on the rates in women than men.

Rates of sepsis were highest among the youngest and oldest age groups in both sexes. The highest rates of sepsis in active component women were seen in those of Hispanic race or ethnicity, marines, enlisted members, and service members in combat-related occupations. The highest rates of sepsis among active component men were seen in those of White, non-Hispanic race or ethnicity, marines, enlisted members, and service members in motor transport and health care occupations.

This study attempted to better understand descriptive characteristics of female ACSMs hospitalized with sepsis. Among co-occurring diagnoses with sepsis, kidney infections such as pyelonephritis and UTIs were the most frequent co-occurring conditions in incident sepsis encounters among women. Hospitalization rates for genitourinary disorders have consistently been higher among females in the active component, with a risk rate difference of 3.6 per 1,000 p-yrs reported in 2019. ^13^ Trends in pyelonephritis and UTIs have not been assessed in the U.S. military population more recently than 2013 ^14^ and may warrant further study. Genitourinary diseases were also the second most frequent reason for a health encounter in the week prior to sepsis diagnosis (n=372) (data not shown).

In 2 large multi-center cohorts (Kaiser Permanente Northern California and Ann Arbor Veterans Health Administration systems) that included over 46,000 patients, 45% of sepsis patients were seen by clinicians in the week preceding hospitalization, with sharp increases just prior to admission. ^15^ In another study of patients in a Michigan Medicine center hospitalized for sepsis, 10% of these patients were seen in a clinic within 1 day of admission. ^16^ In the present study, 70% of individuals had a health care encounter in the week prior to sepsis hospitalization. Early interactions with medical providers can lead to improved outcomes from sepsis infections if symptoms are recognized early in treatment. Sepsis severity could not be assessed in this study, but if individuals who sought care prior to their sepsis diagnoses had lower morbidity and mortality related to sepsis, this could be evidence of good practices in sepsis care and management by providers. Encouraging care-seeking behavior for kidney and UTIs among women may reduce the number of infections that progress to sepsis by providing treatment earlier in the infection. Early identification of signs of sepsis and early interventions help mitigate debilitating long-term effects from infection.

Co-morbid conditions were evaluated as potential risk factors for women. Chronic medical conditions have been shown to be associated with increased risk of sepsis and severity of sepsis due to their effects on the immune system or inflammation. ^10 - 12^ In this study, women with prior history of chronic kidney disease and chronic liver disease had rates 13 times and 8 times, respectively, higher compared to women without prior history of those 2 co-morbidities. Previous population-based studies have shown strong associations between chronic kidney disease and risk of hospital admission and death from sepsis. ^11^ Liver dysfunction is not only a risk factor for sepsis but is also associated with multiple organ dysfunction and death due to sepsis. ^17^ Additional analyses of sepsis outcomes, such as severity of illness and mortality, among women service members with co-morbidities would be needed to better quantify risk.

This study is limited due to its use of administrative health records to identify sepsis hospitalizations. While administrative health records allow for large, population-based studies, it is possible that sepsis is under-coded in administrative health records. ^18^ ICD coding errors are also possible in administrative health records, which may result in misclassification. Due to limitations with casualty data in DMSS, cause of death cannot be determined, and sepsis-related mortality could not be examined fully. Of note, 47 (2.9%) female ACSMs died after sepsis hospitalization, compared to 268 (5.7%) male ACSMs (data not shown). There were no deaths among women with pregnancy-related sepsis diagnoses during the surveillance period. Additional studies may be warranted to examine mortality outcomes related to sepsis in the military population. Treatment received, such as antibiotics or intravenous fluids, could not be quantified in this study.

Further insights into the differing rates of sepsis among female and male ACSMs may be obtained through chart review, which could help address concerns about misclassification of cases due to possible differences in diagnosis patterns by physicians. The underlying cause of sepsis may also provide clues behind the disparities as well if there are differences by sex. For example, if female ACSMs are more likely to develop sepsis from genitourinary sources than respiratory infections, there may be opportunities to promote health education about genitourinary infections, and for increased vigilance in monitoring these types of infections. Ensuring proper treatment and adherence to treatment with antibiotics of UTIs, for instance, may also play a role in reducing the progression to sepsis. Inadequate treatment of UTIs can lead to recurrence and spread of infection. ^19^

The impact of COVID-19 on the decline in sepsis hospitalizations in 2020 remains unexplained. Sepsis rates in both men and women were higher in 2021 and 2022 compared to 2020, suggesting that some aspect of the early pandemic affected sepsis rates. It cannot be determined from this study whether that decline was due to changes in health care seeking-behavior or preventive measures implemented during the pandemic that reduced the prevalence of other infections. It has been noted that severe COVID-19 infections causing organ dysfunction may not have been coded as sepsis, ^20^ which could explain the 2020 decline.

Sepsis has been identified as a key area of focus for the Defense Health Agency (DHA)'s Critical Care and Trauma Clinical Community, which created DHA's Sepsis Working Group in 2021. ^21^ The Sepsis Working Group issued a memorandum in 2025 that established a sepsis strategy in military hospitals and clinics that includes multi-disciplinary teams and a process for monitoring facility performance on sepsis-related quality measures. As efforts are made within the Military Health System to evaluate sepsis treatment and prevention strategies, this study demonstrates the importance of stratifying by sex in the study of sepsis in the military population.

Treatment and intervention strategies to prevent sepsis infections may differ by sex. Care-seeking behavior has also been shown to differ between male and female service members: Women tend to seek care at higher rates than men, ^22^ which may have implications for differences in sepsis severity and mortality outcomes by sex, which could not be assessed in this study. Future work on sepsis in the U.S. military should include measures of severity including sepsis-related mortality and disease severity.
